# Phytochemical profiling and cytotoxic potential of *Arnebia nobilis* root extracts against hepatocellular carcinoma using in-vitro and in-silico approaches

**DOI:** 10.1038/s41598-023-38517-8

**Published:** 2023-07-14

**Authors:** Asia Kiran, Awais Altaf, Muhammad Sarwar, Arif Malik, Tahir Maqbool, Qurban Ali

**Affiliations:** 1 Institute of Molecular Biology and Biotechnology, The University of Lahore, Lahore, 54300 Pakistan; 2 Department of Plant Breeding and Genetics, Faculty of Agricultural Sciences, University of the Punjab, Lahore, Pakistan

**Keywords:** Biochemistry, Cancer

## Abstract

Hepatocellular carcinoma is the fifth most prevalent cancer worldwide. The emergence of drug resistance and other adverse effects in available anticancer options are challenging to explore natural sources. The current study was designed to decipher the *Arnebia nobilis* (*A. nobilis*) extracts for detecting phytochemicals, in-vitro evaluation of antioxidative and cytotoxic potentials, and in-silico prediction of potent anticancer compounds. The phytochemical analysis revealed the presence of flavonoids, phenols, tannins, alkaloids, quinones, and cardiac glycosides, in the ethanol (ANE) and n-hexane (ANH) extracts of *A. nobilis.* ANH extract exhibited a better antioxidant potential to scavenge DPPH, nitric oxide and superoxide anion radicals than ANE extract, which showed better potential only against H_2_O_2_ radicals. In 24 h treatment, ANH extract revealed higher cytotoxicity (IC_50_ value: 22.77 µg/mL) than ANH extract (IC_50_ value: 46.74 µg/mL) on cancer (HepG2) cells without intoxicating the normal (BHK) cells using MTT assay. A better apoptotic potential was observed in ANH extract (49.10%) compared to ANE extract (41.35%) on HepG2 cells using the annexin V/PI method. GCMS analysis of ANH extract identified 35 phytocompounds, from which only 14 bioactive compounds were selected for molecular docking based on druggability criteria and toxicity filters. Among the five top scorers, deoxyshikonin exhibited the best binding affinities of − 7.2, − 9.2, − 7.2 and − 9.2 kcal/mol against TNF-α, TGF-βR1, Bcl-2 and iNOS, respectively, followed by ethyl cholate and 2-Methyl-6-(4-methylphenyl)hept-2-en-4-one along with their desirable ADMET properties. The phytochemicals of ANH extract could be used as a promising drug candidate for liver cancer after further validations.

## Introduction

Cancer is considered a second, and liver cancer is the third most common cause of frequent demise worldwide. The highest liver cancer incidence rate (> 75%) is recorded in Africa and Asia^1^. Annually, more than 750,000 new cases of liver cancer (33,000 in the United States) are reported and overtaken all other malignancies in terms of incidence and mortality^2^. Persistent viral infections, including HBV and HCV, excessive alcohol consumption, and non-alcoholic fatty liver are the most common risk factors which cause chronic inflammation following liver cirrhosis that eventually develops into HCC^3,4^. In recent years, radical therapies, including surgical resection, ablation, and liver transplantation, have improved the prognosis, but non-specificity, adverse effects and recurrence are continuous barriers to HCC treatment^5^. There is an urgent need to explore some natural resources to find novel pharmaceutically active compounds with greater specificity and minimal or no side effects^6^. Therefore, a comprehensive understanding of the underlying mechanism involved in the pathogenesis of this malignancy may be the first step in developing effective and new treatment strategies.

Traditionally, medicinal herbs have been used to manage several human ailments, including malignant tumors^7^. Many in-vivo and in-vitro investigations reported that the natural chemicals exhibited antitumor potential by inhibiting enzymatic activity, promoting DNA repair pathways, improving antioxidant mechanisms, and initiating apoptosis in cancer cells^8^. Most plant sources are still unexplored for their noteworthy ameliorative effect against malignant diseases. Among various approaches, the GCMS technique is important in phytochemical profiling and chemo-systematic evaluation of medicinal plants^9^. Furthermore, computational approaches are thought to be the most successful, economical, and sophisticated ways to predict the most suitable candidates for drug discovery. It also gives valuable information about the underlying therapeutic mechanism of bioactive components involved in the treatment of carcinogenesis^10^. In-silico research is focused on comprehending the behavior of a biologically active compound inside an organism, mainly depending on druggability and ADMET filters; otherwise, it may cause 40% rejections of selected pharmaceuticals at the end of different trials^11,12^.

*Arnebia nobilis* Reichb. f (local name; ratan jot) belongs to the Boraginaceae family, is indigenous to Afghanistan, and is traditionally used to treat various human ailments^13^. Shikonin, alkannin, and isohexenylnaphthazarin ester derivatives are naphthoquinones that are essential constituents of this plant^14^. Numerous researches have demonstrated that Arnebia species offer a wide range of anti-inflammatory, antitumor, alleviating fever, and wound healing properties^15^. However, in different research studies, the antioxidant and anti-ageing potential of *A. nobilis* was investigated^16,17^. Thus, the therapeutic potential of various biomolecules from this plant unveiling the underlying antiapoptotic and anticancer mechanisms remains unexplored.

In recent decades, the concept of inflammation-induced cancer has been established. Studies have revealed that inflammatory mediators affect almost all stages of tumor development and the efficacy of therapies^18^. Elevated levels of inflammatory cytokines were frequently observed in HCC patients and associated with poor prognosis^19^. TNF-α is a key mediator of inflammation, produced by macrophages and a variety of cancer cells, and provides a molecular connection between chronic inflammation and tumor pathogenesis^20^. TNF-α activates the NF-κB pathway to promote cell proliferation, angiogenesis, invasion, metastasis, and inhibition of apoptosis^21^. Anti-TNF-α therapies impeded the progression of HCC tumors by inducing cell death and reducing inflammation by downregulating various pro-inflammatory cytokines, including TNF-α, IL-6, IL-1β and IL17^22^. Also, TNF-α, INF-γ and IL-1β are well-known for the induction of iNOS in macrophages, fibroblasts, and neutrophils via phosphorylating p65/relA unit of NF-κB and activating JAK/STAT1 pathway^23^. It was observed that iNOS inhibitors suppressed the proliferation of cells in the PDX human model of HCC^24^. A previous study showed that the anti-carcinoma effect of *Nigella sativa* was observed by attenuating the iNOS pathway and inflammatory response mediated by TNF-α in HCC^25^. Transforming growth factor (TGF)-β is a multifunctional cytokine that modulates carcinogenesis by stimulating smad and non-smad pathways^26^. At early stages of liver carcinogenesis, TGFβ-1 suppresses the proliferation of cancer cells while promoting EMT, angiogenesis, proliferation, invasion, and metastasis in later stages^27^. TGF-β binds to TGF-βR2, and TGF-βR1 recognizes the binding molecule to form a tetramer complex. After complex formation, TGF-βR2 cross-phosphorylate TGF-βR1, leading to phosphorylation and dimerization of smad proteins, translocated in the nucleus, to activate a number of transcription factors participated in a cascade of biological functions^28^. During the development of pathological fibrosis and carcinogenesis, the importance of TGF-βR1 in the TGF-β signaling pathway is indisputable. Recently, several TGF-βR1 inhibitors in clinical trials have received much attention as a possible anti-HCC target^29^.

Additionally, a recent study has demonstrated that suppressing the activity of TGF-β ameliorates the efficacy of sorafenib during anti-HCC treatment^30^. Another target protein, Bcl-2 has been identified as a new category of oncogenes that encourage carcinogenesis by inhibiting apoptosis but have no effect on cell proliferation^31^. Its overexpression may have a role in modulating cell growth, cell cycle, DNA repair, and chemo-resistance. Abnormal expression of Bcl-2 was observed in several human malignancies, including liver, colon, lung, stomach, prostate, breast cancer, and neuroblastoma^32,33^. Therefore, targeting different pro- and anti-inflammatory cytokines involved in carcinogenesis is one of the important therapeutic approaches to improve hepatocellular carcinoma therapies. To our knowledge, it is the first comprehensive report to predict the anticancer potential of biomolecules against inflammatory cytokines and an apoptotic protein compared to the FDA-approved synthetic drug sorafenib.

Therefore, the present research was planned to evaluate the in-vitro antioxidative, antitumor, and antiapoptotic activities of *A. nobilis* available in Pakistan. The bioactive compounds were identified from ANH extract using the GCMS technique. Further, in-silico methods were applied to unveil the therapeutics of effective and safe phytoconstituents against anti-HCC targets for managing and treating inflammation-dependent hepatocarcinogenesis.

## Materials and methodology

### Extraction of plant materials

The roots of *A. nobilis* were purchased from a local market in Lahore, Pakistan, and verified by a renowned taxonomist, Dr. Zaheer-ud-Din Khan, professor in the Department of Botany at Government college university, Lahore, Pakistan. The assigned voucher number is G.C.Herb.Bot3780 to *A. nobilis* and samples were also submitted to the herbarium bank of the university. The roots (dried) were pulverized using a herb grinder. The plant powder (400 g per 600 mL of solvent) was macerated in ethanol and n-hexane solvents and kept for at least two weeks at 37 °C. The percentage yield of ANE and ANH extracts was found to be 4.64 and 6.55, respectively. The resulting solutions were filtered through filter paper (Whatman No. 1). The remaining solvent was removed using a rotary evaporator, operating at 35–40 °C, then further dried by lyophilizer and stored at − 20 °C for experimentation.

### The reagents

Most of the reagents were purchased from Sigma Aldrich. These reagents included ethanol (99%), n-Hexane, Folin-ciocalteu reagent, ascorbic acid, quercetin, gallic acid, 2,2-diphenyl-1-Picrylhydrazyl, Dulbecco's Modified Eagle Medium (DMEM), Fetal bovine serum (FBS), Dimethyl Sulfoxide (DMSO), and 3-(4,5-dimethylthiazol-2-yl)-2,5-diphenyltetrazolium bromide (MTT).

### Qualitative profiling of phytochemicals

To prepare stock solutions, one gram (1 g) of dried ethanol and n-hexane extract was dissolved in 200 mL of their respective mother solvents to prepare stock solutions. The resulting stock solutions were subjected to qualitative analysis to determine the presence of secondary metabolites/phytochemicals.

#### Determination of flavonoids

Two to three drops of diluted sodium hydroxide were mixed with 1 mL of sample stock solutions, and in turn, the crude extract developed yellow color. When a few drops of mild H_2_SO_4_ was mixed in the reaction mixture, the solution turned colorless, indicating the presence of flavonoids^34^.

#### Determination of alkaloids

The alkaloids in test samples were detected by adding 2–3 drops of Mayer's reagent in 1 mL of each plant extract. The creamy-white precipitates confirmed the presence of alkaloids^35^.

#### Determination of quinones

Each stock solution (1 mL) was treated with concentrated H_2_SO_4_ (1 mL). The formation of red precipitates indicated the presence of quinones^36^.

#### Determination of saponins

In this experiment, 1 mL of each plant extract was diluted with 5 mL of distilled H_2_O, which was manually stirred for 10 min. A layer of foam for saponins was developed on top of the solution in a test tube and persisted even after adding HCl solution^37^.

#### Determination of cardiac glycosides

A drop of FeCl_3_ solution was added after each of the two experimental stock solutions (2 mL) had been treated with 1 mL of glacial acetic acid. Then, 2 mL of concentrated H_2_SO_4_ was added to the reaction mixture. The development of a brown ring was positive for glycosides^37^.

#### Determination of tannins

For qualitative analysis, 3–4 drops of lead acetate were mixed with 1 mL of plant samples. The presence of tannins was observed as positive due to the appearance of the white-brown precipitates^35^.

#### Determination of Phenols

A small amount of plant extracts were treated with 1 mL of distilled water after adding a few drops of FeCl_3_. The emergence of black or bluish color confirmed that phenols were present in plants^36^.

#### Determination of terpenoids

After the 0.5 mL of plant stock solution had been treated with 2 mL of chloroform, 3 mL of concentrated H_2_SO_4_ was carefully added to make the layer. The terpenoid was positive for the development of reddish-brown color^36^.

### In-vitro antioxidative potential of plant extracts

#### DPPH scavenging assay

The antioxidant capabilities of plants were estimated using DPPH (2-diphenyl-1-picryl- hydroxyl) solution^38^. All stock solutions were prepared by dissolving plant extracts (5 mg/mL), ascorbic acid (5 mg/mL), and DPPH (0.004% w/v; 0.004 g in 100 mL) in 95% methanol. Various concentrations of plant extracts (50–250 μg/mL) and standard reagents were prepared by serial dilutions. To perform the DPPH assay, 0.1 mL of each sample was mixed with 3 mL of freshly prepared DPPH solution and kept in a pitch-dark place for 30 min. A control sample was also prepared containing the same volume of DPPH but with 0.1 mL of methanol. After incubation, the absorbance was measured at 517 nm using a spectrophotometer. Lower absorbance of the samples indicated greater activity in scavenging free radicals. The percentage inhibition activity was measured using the formula:

$${\text{Inhibition}}\;{\text{of}}\;{\text{free}}\;{\text{radicals}}\;\left( \% \right) = \left( {{\text{A}}_{{{\text{Control}}}} - {\text{A}}_{{{\text{Sample}}}} } \right)/{\text{A}}_{{{\text{Control}}}} \times 100$$

#### Nitric oxide (NO) scavenging assay

The scavenging capacity of NO (free radical), produced by sodium nitroprusside, was estimated by the method reported by^39^. Each plant extract (1 mL) with varying concentrations (50–250 μg/mL) was mixed with 0.5 mL of sodium nitroprusside (10 mM) and 1 mL of phosphate buffer saline (pH; 7.4), was maintained for 4 h at 25 °C. The testing solution was centrifuged for 5 min at 3000 rpm. After centrifugation, 0.5 mL supernatant was mixed with 0.5 mL of Griess reagent. The absorbance was estimated against blank at 546 nm. The following equation measured the inhibition of free radicals:

$${\text{Scavenging of nitric oxide }}\left( \% \right) \, = \, \left( {{\text{A}}_{{{\text{Control}}}} - {\text{A}}_{{{\text{Sample}}}} } \right)/{\text{A}}_{{{\text{Control}}}} \, \times {1}00$$

#### Superoxide anions scavenging assay

The antioxidant potential of each extract to quench free radicals was estimated using the procedure described by^40^. This reaction mixture contains 0.3 mL of nitroblue tetrazolium (0.5 mM), 0.5 mL of 50 mM of PBS (pH 7.6), 0.3 mL of 50 mM of riboflavin and 1 mL of plant samples, and ascorbic acid with various concentrations (50 to 250 μg/mL). The reaction would start when adding 0.25 mL of phenazine methosulphate (20 mM) solution. The solution was incubated for 20 min at 20 °C. The results of plant extracts were calculated by taking absorbance at 560 nm against a blank. The capability of plant extracts to inhibit the superoxide radicals were estimated using the given equation:

$${\text{Inhibition of superoxide anions }}\left( \% \right) \, = \left( {{\text{A}}_{{{\text{Control}}}} - {\text{A}}_{{{\text{Sample}}}} } \right)/{\text{A}}_{{{\text{Control}}}} \times {1}00$$

#### Hydrogen peroxide (H_2_O_2_) scavenging assay

The standard procedure estimated the scavenging potential of plant extracts with a slight modification^41^. Hydrogen peroxide solution was prepared with a concentration of 2 mM/L in phosphate buffer saline (pH 7.4; 50 mM). Each herbal extract (0.1 mL) with varying concentrations (50 to 250 μg/mL), PBS (0.3 mL), and H_2_O_2_ solution (0.6 mL) were added and left for ten min at room temperature. The absorbance of plant fractions and blank was taken at 230 nm compared with ascorbic acid (standard reagent). The antioxidant potential of plants to quench the free radicals is evaluated by the given formula:

$${\text{Inhibition of hydrogen peroxide }}\left( \% \right) \, = \left( {{\text{A}}_{{{\text{Control}}}} - {\text{A}}_{{{\text{Sample}}}} } \right)/{\text{A}}_{{{\text{Control}}}} \times { 1}00$$

### In-vitro cytotoxic potential of plant extracts

#### Culturing of cell line

Both hepatocellular carcinoma cells (HepG2) and baby hamster kidney cells (BHK) were taken from the Institute of Molecular Biology and Biotechnology (IMBB), The University of Lahore, Pakistan. Both cell lines were grown in DMEM (Dulbecco's Minimum Essential Medium) supplemented with 10% FBS (Fetal bovine serum) and penicillin/streptomycin. Cells were seeded in a cell culture flask (T75) and kept at room temperature with CO_2_ (5%) and air (95%) in an atmospheric chamber^42^.

#### Treated cell line groups

Normal and malignant cells were grown to calculate IC_50_ values, percentage cell viability, and apoptotic induction. Based on treatment, cultured cells were separated into four groups. The first group, which contains only DMEM medium, was labeled as an untreated group (UT) and taken as a negative control. The second and third groups were treated with varying doses of the herbal extracts labeled ANE (ethanol extract of *A. nobilis*) and ANH (n-hexane extract of *A. nobilis*). The fourth group was treated with different doses of cisplatin as a standard drug against cancer and normal cells.

#### Cell counting and cytotoxicity analysis

Using the standard method, the cytotoxic potential of several plant extracts was assessed^43^. For the MTT assay, cells (HepG2 and BHK) were seeded (1 × 10^4^ cells/well) in a 96-well plate and were treated with increasing concentrations (10–100 μg/mL) of both the plant samples and cisplatin while the untreated cells having only medium and kept for twenty-four hrs. Treated and untreated cells were rinsed with PBS (200 μL) to remove the extra medium. MTT reagent (25 μL) in PBS was introduced to each well and kept for 3 h at 37 °C. After removing the MTT dye, the formazan crystals were dissolved using 100% DMSO (150 µL). The absorbance was taken at 570 nm using an ELISA microplate reader (BioTek). Each experiment was performed in triplicates. The concentration (IC_50_) exhibited 50% inhibition of cancer, and normal cells were measured using the non-linear regression method.

#### Morphological examination

Using the Floid Cell Imaging Station, the morphological changes due to the inhibitory effect of various concentrations of sample extracts were observed and examined in HepG2 and BHK cells after comparing with the untreated cells served as a control group^44^.

#### Cell viability analysis via crystal violet assay

The percentage of adherent cells was detected in HepG2 and BHK cells using crystal violet staining^45^. The cells were grown in a 12-wells plate and treated with different concentrations (IC_50_) of crude extracts and cisplatin for 24 h, as calculated in the MTT assay. After treatment, the media was rinsed with PBS solution, and the cultured cells in each well were stained with 0.05 mL of crystal violet dye (0.5%). The plate was incubated for the next ten minutes at room temperature to stain the cell nuclei, and the excess stain was removed using phosphate buffer saline. The treated cells were de-stained with ethanoic acid (10%), and the absorbance of each well was measured at 600 nm. Each experiment was conducted three times independently. The cytotoxic activity was determined using the following formula:

$${\text{Percentage of viable cells }}\left( \% \right) \, = {\text{ Treated cells}}/{\text{Untreated cells }} \times { 1}00$$

#### Muse analysis (Annexin V/PI)

The apoptotic index of plant extracts was detected through the annexin V/PI Assay Kit (Merck-Millipore; Cat. No. MCH100105) according to recommended protocol by the manufacturer^46^. Briefly, both HepG2 and BHK cells (1 × 10^4^ cells per well) were cultured on a 12-wells plate and kept for one day after treatment with plant extracts and cisplatin at IC_50_ concentrations. The cultured cells were taken after centrifugation for 5 min at 1000 rpm, rinsed by phosphate buffer saline, and suspended in 1xbindinding buffer (100 μL). After that, each suspension was stained through the annexin V-FITC binding (5 μL) and propodium iodide (10 μL) and left in a light-restricted area at room temperature for fifteen min. Results of cell death induction were measured using Muse™ (Merck-Millipore) automated cell analyzer. The experiments were conducted independently three times.

### GC/MS-based identification of the plant constituents

For routine compound analysis, gas chromatography-mass spectroscopy is a preferable method. Based on antioxidant and anticancer results, n-hexane extract was found to be more effective as a cytotoxic agent against hepatocellular carcinoma. So, the n-hexane extract was chosen for GC–MS analysis to identify bioactive molecules. For this purpose, n-Hexane extract of *A. nobilis* was injected using the split-less injection mode on a DB-5 MS capillary column covered with polydimethylsiloxane and measuring 30 m × 250 µm × 0.25 µm in size. The GCMS TQQQ Agilent has been outfitted with a QP-5000 (quadrupole) mass spectrometer. Helium (1.5 mL/min) served as the carrier gas, while 70 eV was the ionization voltage used in this experiment. The oven temperature was maintained at 50 °C for 3 min, increasing by 7 °C/min up to 180 °C for 25 min, while a temperature of 250 °C was maintained for the injector port and detector. By using a mass spectrophotometer, it is divided into different constituents with varying retention indices. The software connected to it recorded different peaks against each retention index displayed on the chromatogram. The phytoconstituents were characterized by comparing their mass spectrum to the reference compounds available in NIST-05 library with their known activities and other published mass spectra^47^.

### In silico study

#### Protein preparation

The x-ray crystallography structure of TNF-alpha, TGF-β receptor I kinase, iNOS, and Bcl-2 with PDB IDs of 2az5, Irw8, 4NOS, and 4MAN, respectively, were taken from protein data bank in 3D-PDB format (https://www.rcsb.org). Proteins were prepared by withdrawing the extraneous water molecules and co-crystallized ligands, adding polar hydrogens, Gasteiger charges, and partial charges to the atoms for protonation at physiological pH. The possible active site residues of target proteins were estimated using the CASTp server^48^. For docking, the grid box was set on the co-crystalized ligand, and measurements were recorded in a config.txt file using the AutoDock vina tool, as represented in Table 1. Then, the co-crystallized ligand was removed from the protein and saved in pdbqt format.

**Table 1:** The grid box dimensions for anti-HCC target proteins are given as follows:

Target proteins	Center			Size			Exhaustiveness
	X	Y	Z	X	Y	Z	
TNF-α	− 18.486	72.753	38.929	40	40	40	8
TGF-βR1	7.327	17.295	17.025	40	40	40	8
iNOS	4.035	95.635	20.795	40	40	40	8
Bcl-2	− 11.927	8.043	4.358	40	40	40	8

#### Ligand preparation

For in-silico assessment, the structure of identified natural compounds with anti-HCC potential was downloaded from 'PubChem' or 'ChemSpider’ databases in an SDF format and saved in a PDB format (protein data bank format) using BIOVIA Discovery Studio visualizer^49^. The ligands were prepared by independent uploading into the autodock vina tool. Gasteiger charges and non-polar hydrogen atoms were added, while rotational interactions were determined and changed.

#### Selection of drug-like biomolecules

Pharmacokinetics and physicochemical properties were essential for identifying therapeutically important candidates to act like an effective and safe drug. It was time-consuming and more expensive to analyze these features using in-vitro and in-vivo methods^50^. As a result, we used different in-silico tools to screen and compute these properties of phytochemicals identified from the n-hexane extract of *A. nobilis*. The drug-like compounds were screened based on following Lipinski's "rule of five" and toxicity profiling filter. Lipinski’s characteristics of drug-likeness included molecular weight, lipophilicity, molar refractivity, hydrogen bond acceptors, and donors^51,52^ were estimated using swissADME software (https://www.SwissADME.ch)^53,54^. The toxicology was evaluated by admetSAR (http://lmmd.ecust.edu.cn/) and pkCSM software (http://biosig.unimelb.edu.au/pkCSM/prediction). Mutagenicity, carcinogenicity, and acute oral toxicity parameters were estimated using the admetSAR operating system^55^, while hepatotoxicity was determined by the pkCSM program^56^. SMILES (Simplified molecular input line entry system) of phytocompounds were taken from ‘PubChem’ and entered into the software for corresponding analysis. Any compound that showed any positive sign of toxicity or more than one violation of Lipinski’s rule was removed from this in-silico study.

#### Molecular docking

For molecular docking, the AutoDock vina tool (Graphical User Interface application) was used to prepare the proteins and ligands for saving them in PDBQT format, decrease energies, and set the grid box around active site residues. Utilizing data from grid box values specified in the configuration file and input files of both parameters (proteins and ligands), docking was performed using Lamarckian Genetic Algorithm 4.2^57^. In the docking procedure, macromolecules were taken as rigid entities, while the ligands were flexible to produce different conformations. The lowest favorable binding energy is defined as the stable interactions of the ligand having an RMSD value less than 1 Å with the selected macromolecule. The ligand conformation with the lowest binding affinity was chosen and aligned with the target macromolecule to form a docked complex^58^.

#### Analysis of molecular interactions

The docked complexes were further investigated and visualized for post-dock results analysis using PYMOL © Molecular Graphics (version: 2.5.4, 2010, Shrodinger L.L.C.)^59^, and 2-D and 3-D snapshots were taken by Biovia DiscoveryStudio client 2021. The PLIP web server was used to analyze different molecular interactions involved in the formation of stable ligand–protein complexes^60^.

#### ADMET prediction

The pharmacokinetic analysis of phytocompounds was performed to evaluate their tolerance and safety in human and animal models using various softwares^61^. The pkCSM tool was utilized to predict the detailed investigation of ADMET parameters of best-hit drug-like compounds^56^.

### Ethical statement

It has been confirmed that the experimental data collection complied with relevant institutional, national, and international guidelines and legislation with appropriate permissions from authorities of the Institute of Molecular Biology and Biotechnology, The University of Lahore, Lahore 54300, Pakistan.

## Results

### Qualitative phytochemical profiling

The qualitative phytochemical analysis showed the presence of flavonoids, alkaloids, quinones, cardiac glycosides, tannins, and phenols in both extracts. Terpenoids were present in ANH, while saponin was only identified in ethanolic extract. These compounds are important for exhibiting well-known bioactivities. The phytochemical evaluation of both extracts is given in Table 2.

**Table 2:** Estimation of preliminary phytochemicals in plant extracts of selected medicinal plant.

Sr. no.	Phytoconstituents	ANE (ethanol extract of *A. nobilis*)	ANH (n-hexane extract of *A. nobilis*)
1.	Flavonoids	+	+
2.	Alkaloids	+	+
3.	Quinones	+	+
4.	Saponins	+	−
5.	Cardiac glycoside	+	+
6.	Tannins	+	+
7.	Phenols	+	+
8.	Terpenoids	−	+

(+) indicates the presence of phytocompounds, (−) indicates the absence of phytocompounds.

### In-vitro antioxidant potentiality of plant extracts

In-vitro model was designed to predict the scavenging capability of the ethanolic and n-hexane extracts of *A. nobilis* and a reference reagent (ascorbic acid).

#### DPPH scavenging activity

**Figure 1:**
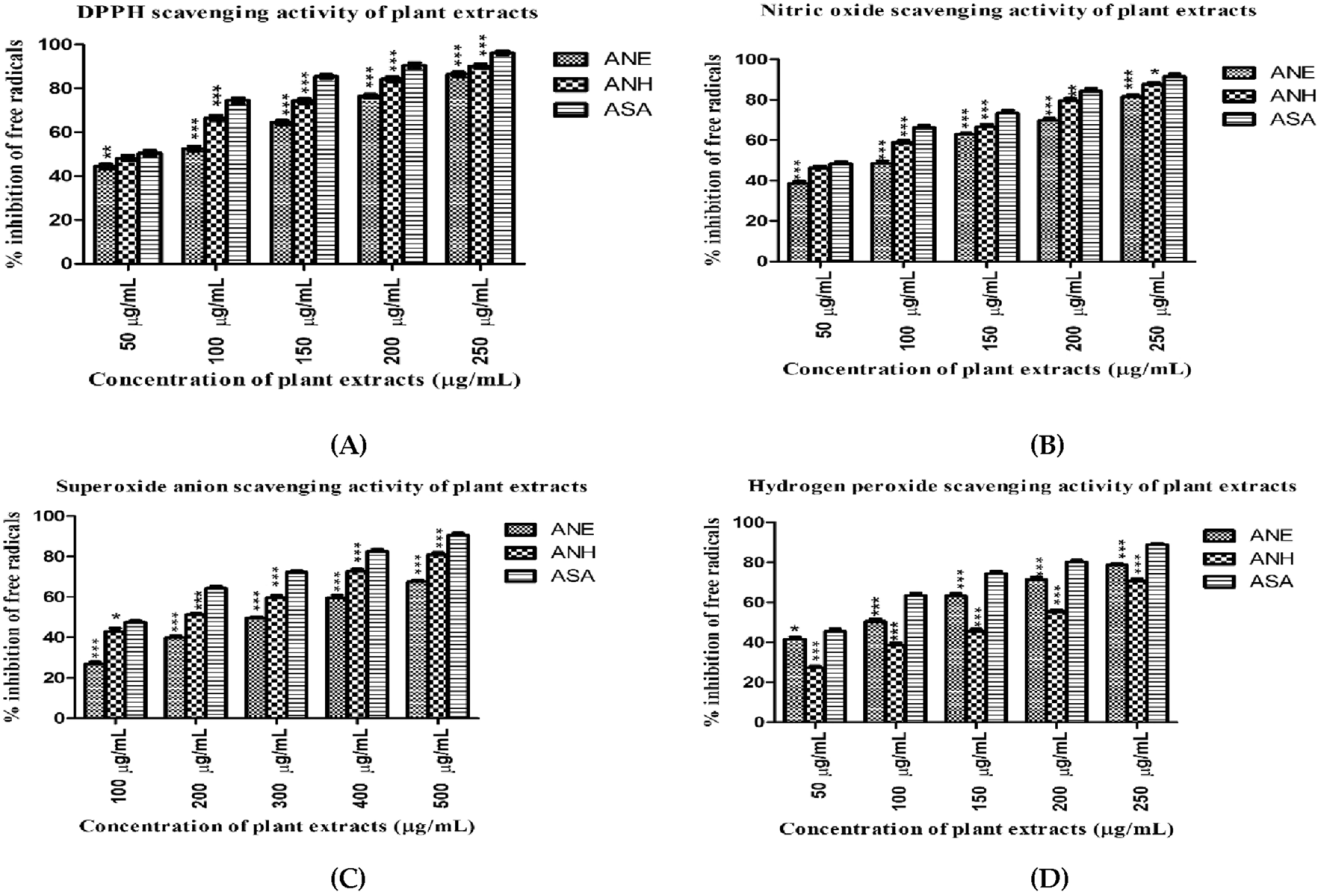
Antioxidant potential of ethanol and n-hexane extract of *A. nobilis* (**A**) DPPH radical scavenging assay (**B**) Nitric oxide radical scavenging assay (**C**) Superoxide anions radical scavenging assay (**D**) H_2_O_2_ radical scavenging assay. All results (n = 3) are significant with the *p*-value ≤ 0.05(***). ANE (ethanol extract of *A. nobilis*), ANH (n-hexane extract of *A. nobilis*), and ASA (ascorbic acid).

The antioxidative capacity of both extracts of *A. nobilis* was calculated. The % inhibition of free radicals scavenging is displayed in Fig. 1A and Table 3. The higher antioxidant potential was observed in ANH extract (IC_50_ = 39.45 µg/mL), and the least potential was detected in ethanol extract (IC_50_ = 81.13 µg/mL) in comparison to the standard reagent (IC_50_ = 13.28 µg/mL).

**Table 3:** The IC_50_ values of both plant extracts and reference reagents against several in-vitro antioxidant models.

Sr. no.	Antioxidant parameters	IC_50_ values in µg/mL (mean ± SD.)		
		ASA	ANE	ANH
1.	DPPH	13.28	81.13	39.45
2.	Nitric oxide	41.16	102.22	64.44
3.	Superoxide anion	47.33	156.75	90.88
4.	Hydrogen peroxide	50.83	91.27	162.10

ANE: ethanolic extract of *A. nobilis*; A.N.H.: n-hexane extract of *A. nobilis;* A.S.A.: ascorbic acid; DPPH: 2-diphenyl-1-picryl-hydroxyl; IC_50_: half-maximal inhibitory concentration.

#### Nitric oxide scavenging activity

The efficiency of plant extracts to quench the nitric oxide radicals was increased significantly (*p*-value ≤ 0.05) with increasing doses, given in Fig. 1B and Table 3. In both plant extracts, ANH showed a better free radical quenching potential with the IC_50_ of 64.44 μg/mL than ANE (IC_50_: 102.22 µg/mL) and the ascorbic acid (41.16 μg/mL). The increasing order of IC_50_ values with decreasing order of antioxidant potential was observed in plants and standard: ASA < ANH < ANE. The antioxidant potential of plant extracts against free radicals may be due to various bioactive compounds.

#### Superoxide anion (O_2_^⋅−^) scavenging activity

All plant extracts and ascorbic acid showed linear dose-dependent moderate scavenging activities (Fig. 1C). The IC_50_ values of ANE, ANH, and positive control (ASA) were observed to be 156.75, 90.88, and 47.33 µg/mL, respectively, as depicted in Table 3. The inhibition of superoxide anion radical of testing samples and a reference reagent was in the following order: ASA > ANH > ANE.

#### Hydrogen peroxide (H_2_O_2_) scavenging activity

H_2_O_2_ is not a toxic compound, but sometimes it becomes reactive and may lead to the accumulation of hydroxyl radicals within the cell. The hydrogen peroxide scavenging assay also evaluated the antioxidant capacity, as shown in Fig. 1D. Among all plant extracts, a lower IC_50_ value (91.27 µg/mL) of ANE revealed its strongest scavenging activity with 41.72–78.69% inhibition of free radicals compared to ANH exhibiting 27.12–70.76% inhibition of radicals with the IC_50_ concentration of 162.10 µg/mL. For comparison, ascorbic acid inhibits free radicals (45.49–88.85%) with the IC_50_ value of 50.83 µg/mL (Table 3).

### In-vitro antitumor activity

#### Calculation of IC_50_ values and cytotoxicity potential

**Figure 2:**
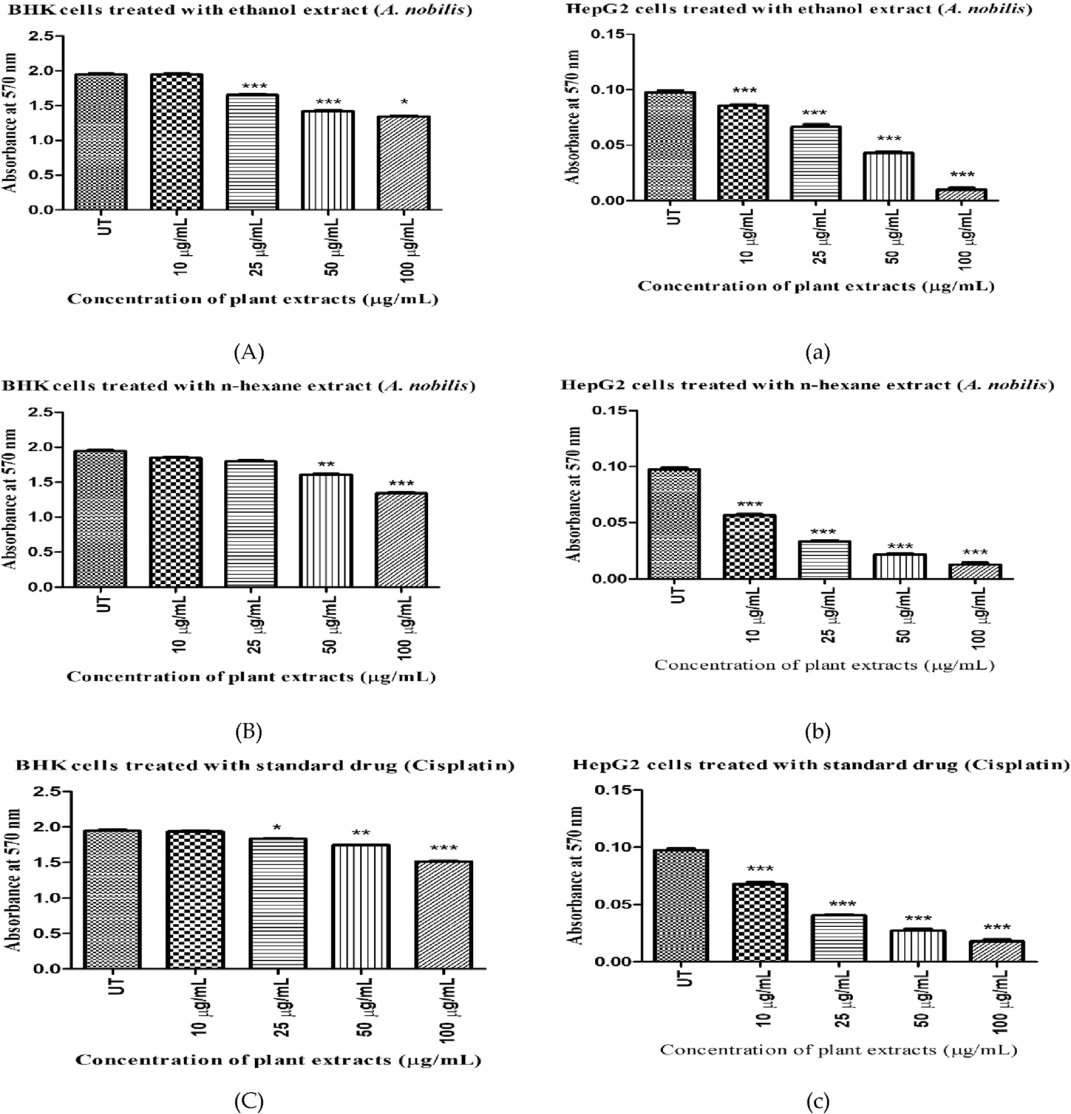
Cytotoxicity of various plant extracts and cisplatin against BHK (Baby hamster kidney fibroblasts) and HepG2 (human hepatoma G2) cells via MTT assay. UT: untreated cells. (**A**) BHK-treated with ANE, (b) HepG2-treated with ANE, (**B**) BHK-treated with ANH, (b) HepG2-treated with ANH, (**C**) BHK-treated with Cisplatin, (c) HepG2-treated with Cisplatin. All results (n = 3) are significant with the *p*-value ≤ 0.05(***). UT: untreated cells, ANE: ethanol extract of *A. nobilis*, ANH: n-hexane extract of *A. nobilis.*

The antiproliferative effects of all plant extracts (10–100 µg/mL) on HepG2 and BHK cell lines were determined using an MTT assay. The results indicated that increasing the concentration of extracts and cisplatin decreased the number of viable cells by inducing more cytotoxicity compared to the untreated cells (Fig. 2). Although, this effect was more prominent in the n-hexane extract with less IC_50_ value (22.77 µg/mL) than its ethanol extract with the IC_50_ concentration of 46.74 µg/mL, while the minimal or no toxicity effect was observed in BHK cells after treatment with both the plant extracts (ANE: IC_50_ = 215 µg/mL and ANH: IC_50_ = 183 µg/mL). Moreover, the conventional chemotherapeutic drug (cisplatin) showed more cytotoxicity with the IC_50_ value of 25.83 μg/mL against HepG2 cells but also proved a bit more toxic towards the normal cells (IC_50_ = 57.22 µg/mL) in contrast to plant extracts.

#### Morphological observations

**Figure 3:**
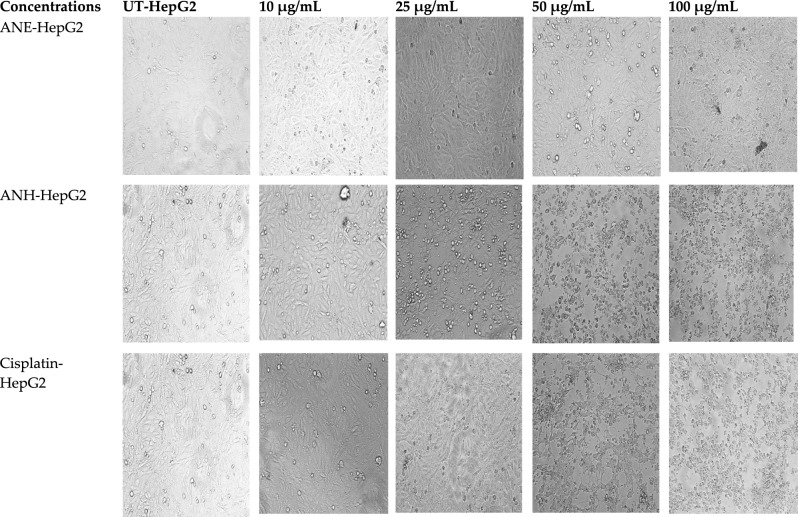
Morphological representation of HepG2 cells with increasing concentrations of plant extracts and cisplatin. Alterations in the morphology of hepatocellular cells in n-hexane and cisplatin-treated cells were obvious at concentrations ≥ 25 µg/mL, while in ethanolic extract, changes were noticeable at ≥ 50 µg/mL. UT: untreated cells, ANE: ethanol extract of *A. nobilis*, ANH: n-hexane extract of *A. nobilis.* All the images were taken at ×20 magnification.

Morphological changes of *A. nobilis* root extracts (ethanol and n-hexane) against HepG2 are shown in Fig. 3. Alterations in the shape, size, and structure of neoplastic cells were estimated in a dose-reliant manner. Most malignant cells lose their ability to adhere to the surface and normal morphology at doses ≤ 50 µg/mL. The MTT assay could not detect any change in cell shape or decrease in the number of HepG2 cells at the lower doses (10 and 25 µg/mL) using ethanolic extract as compared to untreated cells. But cisplatin and n-hexane-treated HepG2 cells showed less viability with degenerated cell shapes at 25, 50, and 100 µg/mL concentrations.

#### Cell viability assessment through crystal violet staining

**Figure 4:**
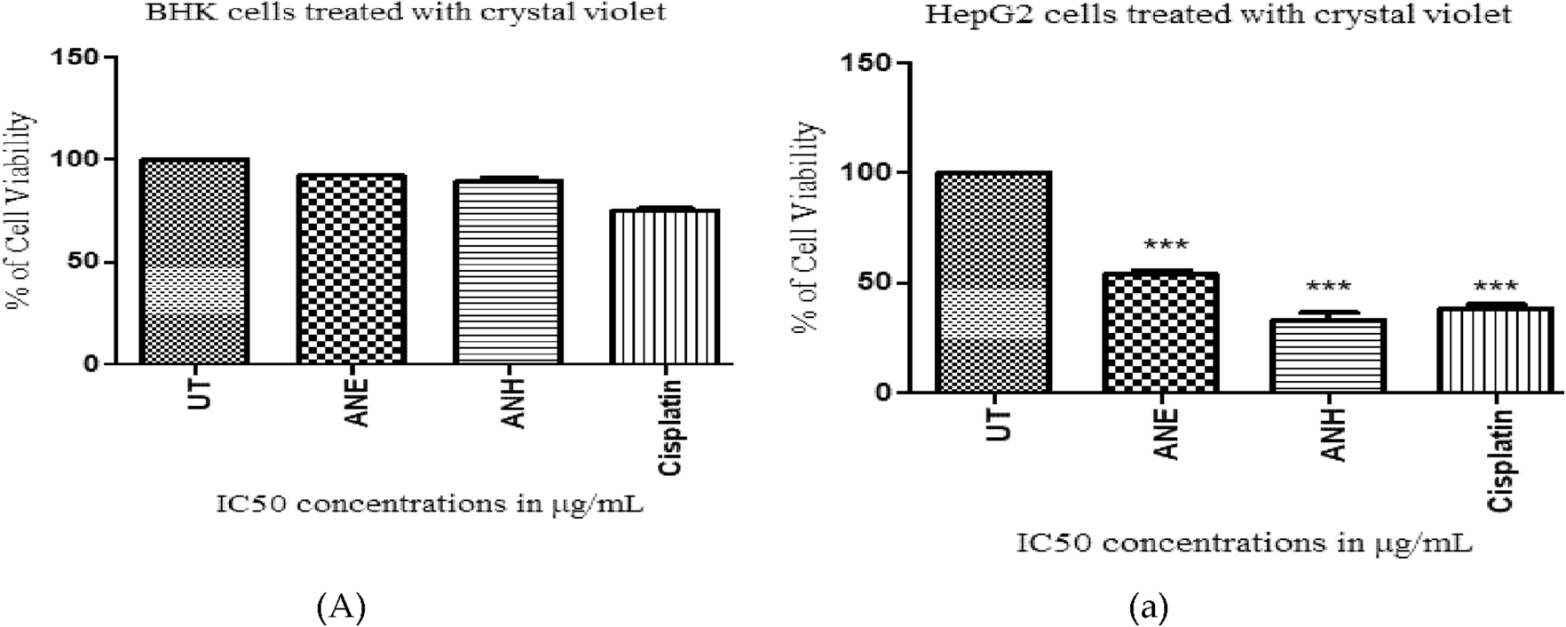
Cell viability assessment through crystal violet staining. (**A**) BHK-treated cells, (**B**) HepG2-treated cells, UT: untreated cells in BHK and HepG2 cell lines. All results (n = 3) are significant with the *p*-value ≤ 0.05(***). ANE: ethanol extract of *A. nobilis*, ANH: n-hexane extract of *A. nobilis.*

The percentage of live cells was evaluated through crystal violet staining in HepG2 (cancerous) and BHK (normal) cells. The plant extracts and reference drug exhibited different responses in a concentration-dependent manner against both cells (Fig. 4A,B). HepG2 cells treated with IC_50_ concentrations of ethanol extract, n-hexane extract, and cisplatin showed 53.82%, 33.35%, and 38.58% of cell viability. The results indicated that the n-hexane extract proved more toxic to cancer cells than ethanol extract, reflecting their higher apoptotic activity by decreasing the number of live cells in HepG2 cells. No significant toxicity was noticed in healthy cells when treated with plant extracts, while more toxicity was observed in cisplatin-treated normal cells. No prominent dead cells were detected in untreated HepG2 and BHK cells.

#### Muse analysis via Annexin V/PI

**Figure 5:**
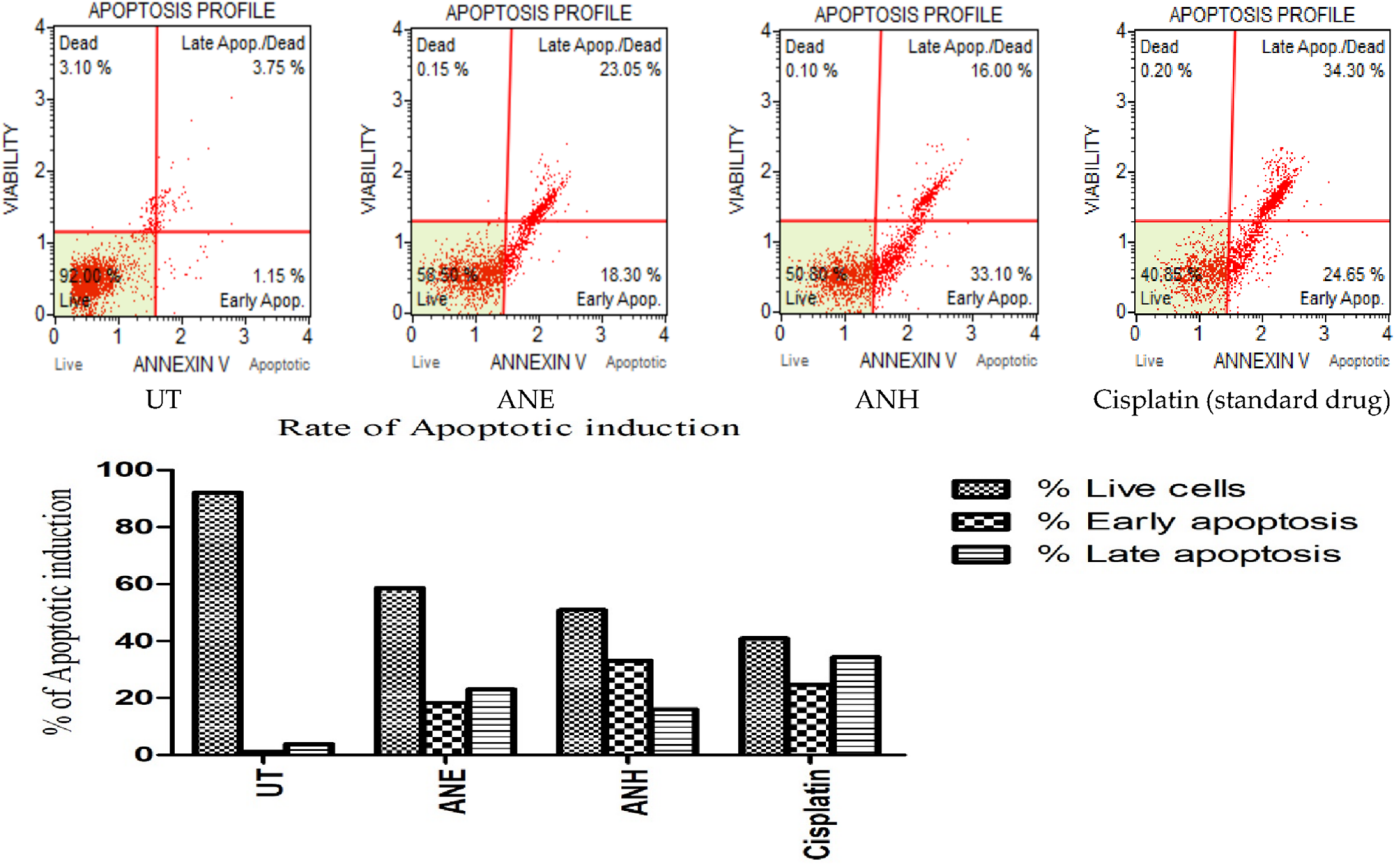
Estimating the percentage of apoptotic induction of plant extracts and cisplatin using HepG2 cells. UT: untreated cells, ANE: ethanol extract of *A. nobilis*, ANH: n-hexane extract of *A. nobilis.*

The apoptotic potential of plant extracts was determined by staining HepG2 cells with annexin V-FITC/PI stains. The apoptotic induction in HepG2 cells treated with plants and cisplatin is depicted in Fig. 5. In the ethanolic and n-hexane extract of *A. nobilis*, the percentages of apoptosis observed in HepG2 cells were 41.35% and 49.10%, respectively. The percentages of apoptosis were observed to be 58.95% when treated with IC_50_ concentration of cisplatin. Compared to the untreated cells (negative control), the hexane-treated groups exhibited a higher percentage of dead cells in the early and late apoptotic stages, reflecting their higher potential of inducing apoptosis than ethanol-treated HepG2 cells. In the case of untreated cells, 92% of viable cells were detected, while after incubation, 1.15% and 3.75% of dead cells were observed in early and late apoptotic profiles, respectively.

### GCMS evaluation of phytochemicals in *A. nobilis*

**Figure 6:**
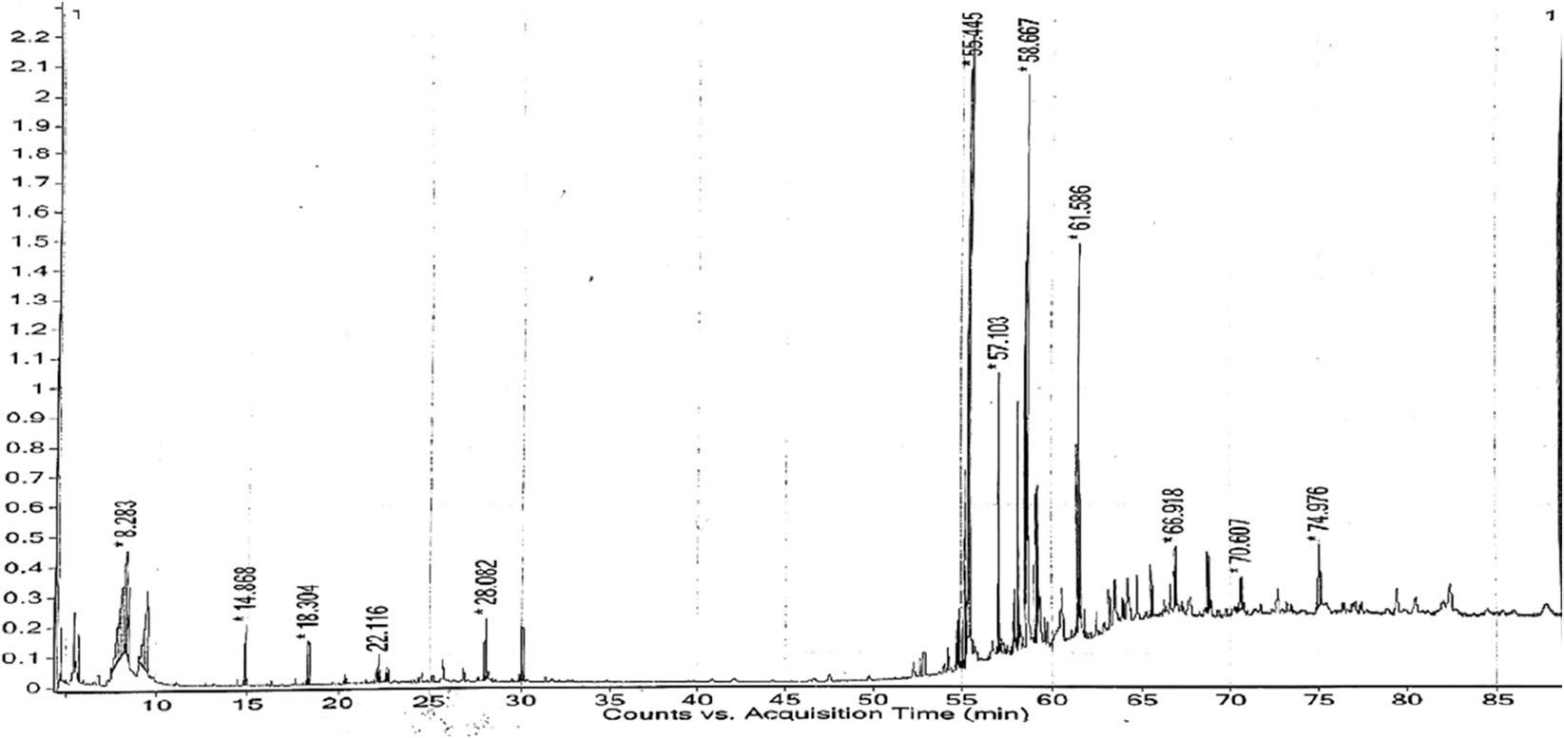
GC–MS chromatogram of n-hexane extract of *A. nobilis.*

A total of thirty-five natural compounds were identified by the GCMS analysis of the n-hexane fraction of *A*. *nobilis* roots exhibited various pharmacological activities, and its chromatogram is presented in Fig. 6. The complete identification of bioactive compounds was made by comparing their mass spectra with retention indices (RI), molecular formula (MF), molecular weight (MW), and percentage concentrations (%) in ANH with the recognized compounds suggested by the NIST 05 library (Table 4). The following compounds were present in the n-hexane fraction of *A. nobilis* roots: Deoxyshikonin, Isovaleric acid, 1,1′-2,2′-Bis[2,3-dimethylbenzoquinonyl], 3,3-Dimethylacrylic acid, 2-(3,7-Dimethyl-octa-2,6-dienyl)-1,4-dimethoxy-benzene, 3-Hydroxy-1-methoxyanthraquinone, alpha-Bergamotene, 5,8-Dihydroxy-1,4-naphthoquinone, Thymol, 6,7-Dimethyl-1H-pyrrolo[3,4-c]pyridine-1,3,4(2H,5H)-trione, 9,12-Octadecadienoic acid, ethyl ester, Phenol,2-methyl-5-(1,2,2-trimethylcyclopentyl), Diisooctyl phthalate, beta-sitosterol and butyl oleate.

**Table 4:** GCMS-based phytochemical characterization of *A. nobilis* (n-hexane).

Sr. no.	Name of phytocompounds	Molecular formula	Molecular weight	Retention index (RI.)	Area (%)	Area sum (%)
1.	Methylcyclohexane	C_7_H_14_	98	781	3.31	0.61
2.	2,2-Dimethoxybutane	C_6_H_14_O_2_	118	685	3.11	0.58
3.	Cyclobutene,2-propenylidene	C_7_H_8_	92	735	2.75	0.51
4.	Isovaleric acid	C_5_H_10_O_2_	102	811	56.85	10.54
5.	2-Methylbutanoic acid	C_5_H_10_O_2_	102	811	17.64	3.27
6.	3,3-Dimethylacrylic acid	C_5_H_8_O_2_	100	860	34.23	6.35
7.	Beta-Hydroxyisovaleric acid	C_5_H_10_O_3_	118	966	9.45	1.75
8.	Thymol	C_10_H_14_O	150	1262	2.93	0.54
9.	Tetradecane	C_14_H_30_	198	1413	0.57	0.11
10.	alpha-Curcumene	C_15_H_22_	202	1524	1.74	0.32
11.	Pentadecane	C_15_H_32_	212	1512	0.98	0.18
12.	2,4-Di-tert-butylphenol	C_14_H_22_O	206	1555	0.85	0.16
13.	alpha-Bergamotene	C_15_H_24_	204	1430	0.77	0.14
14.	5,8-Dihydroxy-1,4-naphthoquinone	C_10_H_6_O_4_	190	1808	3	0.56
15.	2-Methyl-6-(4-methylphenyl)hept-2-en-4-one	C_15_H_20_O	216	1660	1.76	0.33
16.	6,7-Dimethyl-1H-pyrrolo[3,4-c]pyridine-1,3,4(2H,5H)-trione	C_9_H_8_N_2_O_3_	192	1679	9.19	1.7
17.	Phenol,2-methyl-5-(1,2,2-trimethylcyclopentyl)	C_15_H_22_O	218	1776	9.33	1.73
18.	9,12-Octadecadienoic acid, ethyl ester	C_20_H_36_O_2_	308	2193	3.68	0.68
19.	9-Octadecenoic acid, ethyl ester	C_20_H_38_O_2_	310	2185	5.25	0.97
20.	Butyl 9-hexadecenoate	C_20_H_38_O_2_	310	2185	4.7	0.87
21.	Butyl palmitate	C_20_H_40_O_2_	312	2177	18.29	3.39
22.	Deoxyshikonin	C_16_H_16_O_4_	272	2504	100	18.54
23.	2-(3,7-Dimethyl-octa-2,6-dienyl)-1,4-dimethoxy-benzene	C_18_H_26_O_2_	274	2037	30.27	0.61
24.	Butyl linoleate	C_22_H_40_O_2_	336.6	2391	3.08	0.57
25.	Oleic acid	C_18_H_34_O_2_	282	2144	3.32	0.61
26.	3-Hydroxy-1-methoxyanthraquinone	C_15_H_10_O_4_	254	2366	31.07	5.76
27.	Butyl oleate	C_22_O_42_O_2_	338	2383	31.27	5.8
28.	Butyl stearate	C_22_H_44_O_2_	340	2375	6.52	1.21
29.	2,3-Dimethoxyanthracene-9,10-dione	C_16_H_12_O_4_	268	2334	22.27	4.13
30.	Diisooctyl phthalate	C_24_H_38_O_4_	390	2704	13.47	2.5
31.	1,1′-2,2′-Bis[2,3-dimethylbenzoquinonyl]	C_16_H_16_O_4_	272	2251	40.91	7.58
32.	Heptacosane	C_27_H_56_	380	2705	4.97	0.11
33.	Ethyl iso-cholate	C_26_H_44_O_5_	436	3094	3.84	0.71
34.	Octadecane,3-ethyl-5-(2-ethylbutyl)	C_26_H_54_	366	2413	7.98	1.48
35.	Beta-sitosterol	C_29_H_50_O	414	2731	2.65	2.65

### In-silico study

#### Selection of drug-like compounds

Any compound having more than one violation of Lipinski’s rule and showing any positive result for selected toxicological parameters was excluded from the in-silico study (Table 5). Out of thirty-five compounds identified by GCMS analysis, only fourteen compounds were filtered on this criteria and subjected to molecular docking for evaluation of anticancer potential.

**Table 5:** Lipinski’s parameters and toxicity profiling of all compounds identified from the n-hexane extract of *A. nobilis.*

Bioactive compounds	MW^a^	HBA^b^	HBD^c^	LogP^d^	M.R^e^	L.V^f^	Mutagenicity	Carcinogenicity	Hepatotoxicity	Acute oral toxicity
Methylcyclohexane	98.19	0	0	2.90	33.65	Yes, 0 vio	None	None	None	III
2,2-Dimethoxybutane	118.17	2	0	1.32	33.16	Yes, 0 vio	None	None	None	III
Cyclobutene,2-propenylidene	92.14	0	0	2.13	32.23	Yes, 0 vio	None	Yes	No	II
Isovaleric acid	102.13	2	1	0.98	27.92	Yes, 0 vio	None	None	No	III
2-Methylbutanoic acid	102.13	2	1	0.97	27.92	Yes, 0 vio	None	None	No	III
3,3-Dimethylacrylic acid	100.12	2	1	0.89	27.45	Yes, 0 vio	None	None	No	III
Beta-Hydroxyisovaleric acid	118.13	3	2	0.11	29.12	Yes, 0 vio	None	None	No	III
Thymol	150.22	1	1	2.32	48.01	Yes, 0 vio	None	None	Yes	III
Tetradecane	198.39	0	0	4.32	69.41	Yes, 1 vio	None	None	No	III
alpha-Curcumene	202.34	0	0	3.50	69.55	Yes, 1 vio	None	Yes	No	III
Pentadecane	212.41	0	0	4.50	74.22	Yes, 1 vio	None	None	No	III
2,4-Di-tert-butylphenol	206.32	1	1	3.08	67.01	Yes, 0 vio	None	None	No	III
alpha-Bergamotene	204.35	0	0	3.14	68.78	Yes, 1 vio	None	None	No	III
5,8-Dihydroxy-1,4-naphthoquinone	190.15	4	2	1.52	48.29	Yes, 0 vio	Yes	None	Yes	II
2-Methyl-6-(4-methylphenyl)hept-2-en-4-one	216.32	1	0	3.12	69.75	Yes, 0 vio	None	None	No	III
6,7-Dimethyl-1H-pyrrolo[3,4-c]pyridine-1,3,4(2H,5H)-trione	192.12	3	2	0.65	52.58	Yes, 0 vio	None	None	No	III
Phenol,2-methyl-5-(1,2,2-trimethylcyclopentyl)	218.33	1	1	2.72	69.55	Yes, 0 vio	None	None	No	III
9,12-Octadecadienoic acid, ethyl ester	308.5	2	0	5.03	98.59	Yes, 1 vio	None	None	No	III
9-Octadecenoic acid, ethyl ester	310.51	2	0	5.03	99.06	Yes, 1 vio	None	None	No	III
Butyl 9-hexadecenoate	310.5	2	0	5.30	99.06	Yes, 1 vio	None	None	No	III
Butyl palmitate	312.53	2	0	5.39	99.54	Yes, 1 vio	None	None	No	III
Deoxyshikonin	272.30	4	2	2.72	76.66	Yes, 0 vio	None	None	No	III
2-(3,7-Dimethyl-octa-2,6-dienyl)-1,4-dimethoxy-benzene	274.40	2	0	4.11	86.71	Yes, 0 vio	None	None	No	III
Butyl linoleate	336.55	2	0	5.68	108.21	Yes, 1 vio	None	None	No	III
Oleic acid	282.46	2	1	4.27	89.94	Yes, 1 vio	None	None	No	IV
3-Hydroxy-1-methoxyanthraquinone	254.42	4	1	1.71	68.26	Yes, 0 vio	Yes	None	No	II
Butyl oleate	338.57	2	0	5.42	108.68	Yes, 1 vio	None	None	No	III
Butyl stearate	340.58	2	0	5.66	109.15	Yes, 1 vio	None	None	No	III
2,3-Dimethoxyanthracene-9,10-dione	268.26	4	0	2.46	72.73	Yes, 0 vio	Yes	None	No	III
Diisooctyl phthalate	390.56	4	0	5.42	116.30	Yes, 1 vio	None	Yes	No	IV
1,1′-2,2′-Bis[2,3-dimethylbenzoquinonyl]	272.30	4	0	1.74	72.54	Yes, 0 vio	None	None	No	II
Heptacosane	380.73	0	0	7.32	131.39	Yes, 1 vio	None	None	No	III
Ethyl cholate	436.62	5	3	3.99	122.89	Yes, 0 vio	None	None	No	III
Octadecane,3-ethyl-5-(2-ethylbutyl)	366.71	0	0	6.23	127.10	Yes, 1 vio	None	None	No	III
Beta-sitosterol	414.71	1	1	4.79	133.23	Yes, 1 vio	None	None	No	I

^a^Molecular weight ≤ 500**;** ^b^hydrogen bond acceptor ≤ 10**;** ^c^hydrogen bond donor ≤ 5**;** ^d^LogP ≤ 5**;** ^e^Molar refractivity (40–130)**;** ^f^Lipinski’s violations.

#### Molecular Docking analysis for hepatocellular carcinoma inhibitors

**Figure 7:**
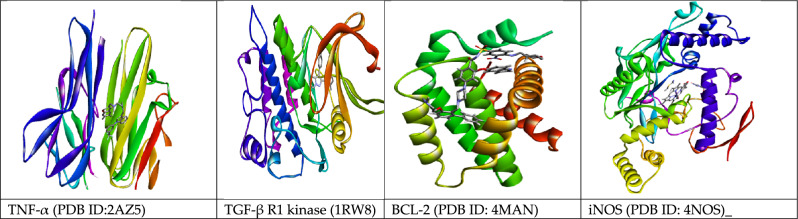
3-Dimensional structures of anti-HCC target proteins.

**Figure 8:**
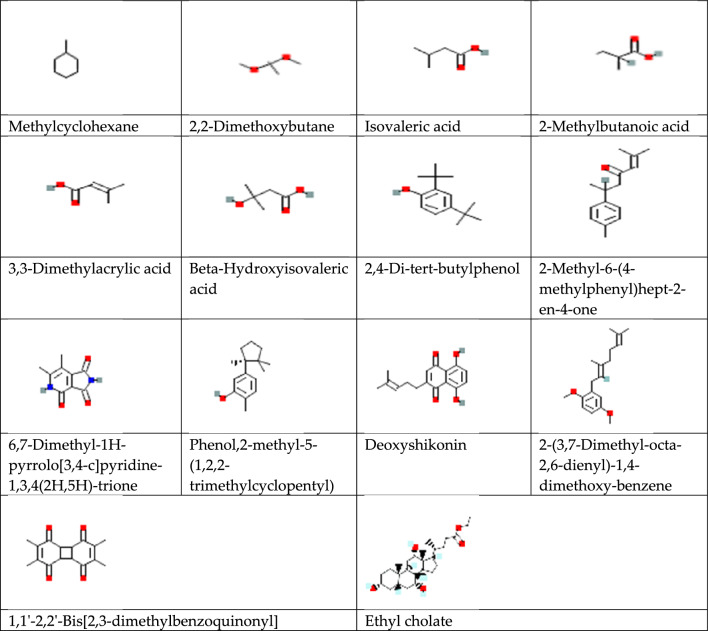
2-Dimensional structure of fourteen phytocompounds selected for molecular docking.

**Figure 9:**
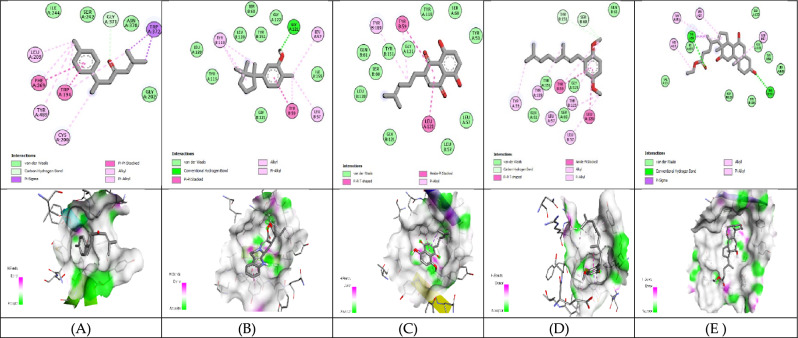
Molecular docking of selected compounds against TNF-α. 2D and 3D structures of docked complexes of phytocompounds, (**A**) 2-Methyl-6-(4-methylphenyl)hept-2-en-4-one, (**B**) Phenol,2-methyl-5-(1,2,2-trimethylcyclopentyl), (**C**) Deoxyshikonin, (**D**) 2-(3,7-Dimethyl-octa-2,6-dienyl)-1,4-dimethoxy-benzene, (**E**) Ethyl cholate.

**Figure 10:**
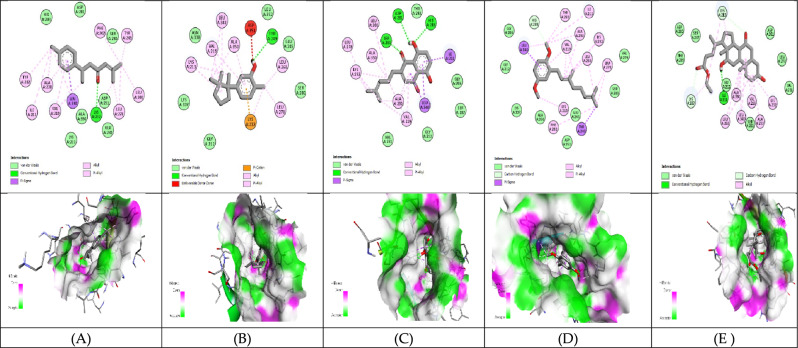
Molecular docking of selected compounds against TGF-βR1. 2D and 3D structures of docked complexes of phytocompounds, (**A**) 2-Methyl-6-(4-methylphenyl)hept-2-en-4-one, (**B**) Phenol,2-methyl-5-(1,2,2-trimethylcyclopentyl), (**C**) Deoxyshikonin, (**D**) 2-(3,7-Dimethyl-octa-2,6-dienyl)-1,4-dimethoxy-benzene, (**E**) Ethyl cholate.

**Figure 11:**
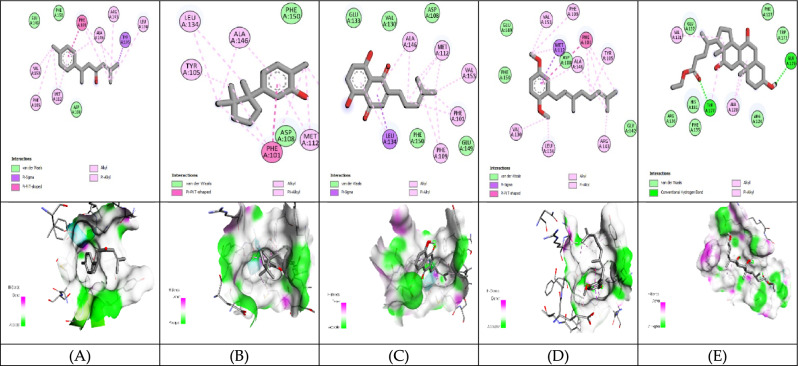
Molecular docking of selected compounds against Bcl-2. 2D and 3D structures of docked complexes of phytocompounds, (**A**) 2-Methyl-6-(4-methylphenyl)hept-2-en-4-one, (**B**) Phenol,2-methyl-5-(1,2,2-trimethylcyclopentyl), (**C**) Deoxyshikonin, (**D**) 2-(3,7-Dimethyl-octa-2,6-dienyl)-1,4-dimethoxy-benzene, (E) Ethyl cholate.

**Figure 12:**
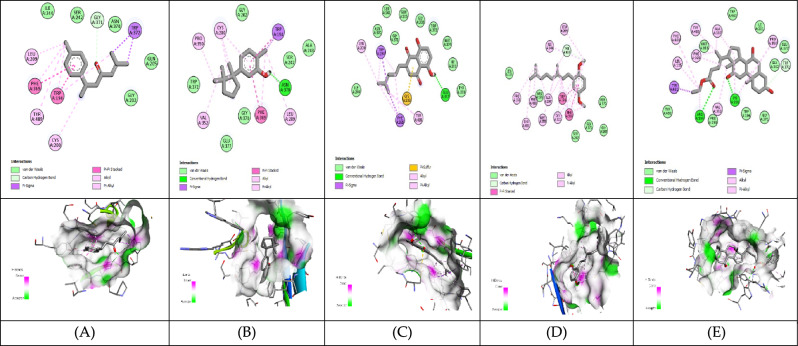
Molecular docking of selected compounds against iNOS. 2D and 3D structures of docked complexes of phytocompounds, (**A**) 2-Methyl-6-(4-methylphenyl)hept-2-en-4-one, (**B**) Phenol,2-methyl-5-(1,2,2-trimethylcyclopentyl), (**C**) Deoxyshikonin, (**D**) 2-(3,7-Dimethyl-octa-2,6-dienyl)-1,4-dimethoxy-benzene, (**E**) Ethyl cholate.

The 3D structure of target proteins and the 2D structure of selected phytocompounds were depicted in Figs. 7 and 8, respectively. Among selected compounds, only five compounds including 2-Methyl-6-(4-methylphenyl)hept-2-en-4-one, phenol,2-methyl-5-(1,2,2-trimethylcyclopentyl), deoxyshikonin, 2-(3,7-Dimethyl-octa-2,6-dienyl)-1,4-dimethoxy-benzene and ethyl cholate showed better docking scores against target proteins and had the potential to develop into an anticancer drug, as shown in Table 6. The information on the interacting amino acid residues involved in hydrogen and hydrophobic bond formations with their bond lengths is represented in Table 7. For TNF-α, the binding affinities of these compounds were between − 6.9 to − 7.8 kcal/mol as compared to the standard drug (sorafenib) with a binding affinity of − 7.7 kcal/mol. The ligand ethyl cholate with the lowest binding affinity formed H-bond with TyrA59 and TyrB151residues and surpassed the standard drug in terms of binding affinity (Fig. 9E). In case of TGF-β R1 protein, 2-Methyl-6-(4-methylphenyl)hept-2-en-4-one, phenol,2-methyl-5-(1,2,2-trimethylcyclopentyl), deoxyshikonin, 2-(3,7-Dimethyl-octa-2,6-dienyl)-1,4-dimethoxy-benzene and ethyl cholate had the lowest binding affinity of − 8.0, − 7.8, − 9.1, − 8.1 and − 7.4, respectively, while the binding energy of sorafenib was − 7.9 kcal/mol. Deoxyshikonin, with the highest docking scores, was found to form three H-bonds with SerA280, HisA283, and AspA281 residues of the target protein (Fig. 10C). For Bcl-2, the binding affinities of the top five compounds were estimated between − 6.8 and − 7.3 kcal/mol as compared to sarafenib (− 7.6 kcal/mol). All compounds have shown better binding energies against these target proteins but have been found to be less effective than standard drugs. Among these compounds, only ethyl cholate formed two H-bonds with Bcl-2 using GluA176 and TyrA177 residues (Fig. 11E). Similarly, the five top scorers compounds showed binding affinities between − 7.4 and − 9.2 kcal/mol against iNOS as compared to sarafenib, with a docking score of − 8.4 kcal/mol. All compounds have shown better results than the control drug except phenol,2-methyl-5-(1,2,2-trimethylcyclopentyl). In 2-D analysis, the best-hit molecule, deoxy-shikonin formed an H-bond via GluA377 residue with a target receptor (Fig. 12C). Among the top five bioactive compounds, deoxyshikonin exhibited a strong inhibitory effect against most of the cancer-causing targets, followed by ethyl cholate and 2-Methyl-6-(4-methylphenyl)hept-2-en-4-one. Overall, all these compounds had a great potential to develop into cytotoxic drugs for treating liver carcinoma. The 3-D and 2-D models of all docked complexes are given in Figs. 9, 10, 11 and 12.

**Table 6:** Binding energies of selected druglike-compounds taken from an n-hexane extract of *A. nobilis.*

Compound’s name	TNF-α	TGF-β R1	Bcl-2	iNOS
Methylcyclohexane	− 4.4	− 5	− 7	− 5.8
2,2-Dimethoxybutane	− 4	− 4.2	− 3.9	− 4.2
Isovaleric acid	− 4.1	− 4.9	− 4.4	− 4.5
2-Methylbutanoic acid	− 4.2	− 5.1	− 4.4	− 4.6
3,3-Dimethylacrylic acid	− 4.2	− 4.7	− 4.2	− 5.0
Beta-Hydroxyisovaleric acid	− 4.5	− 4.9	− 4.3	− 4.4
2,4-Di-tert-butylphenol	− 6.5	− 7	− 6.4	− 7.1
**2-Methyl-6-(4-methylphenyl)hept-2-en-4-one**	− 6.9	− 8.0	**− 7.3**	− 8.6
6,7-Dimethyl-1H-pyrrolo[3,4-c]pyridine-1,3,4(2H,5H)-trione	− 6.0	− 7.2	− 6.2	− 7.3
**Phenol,2-methyl-5-(1,2,2-trimethylcyclopentyl)**	− 7.6	− 7.8	− 6.8	− 7.4
**Deoxyshikonin**	− 7.2	**− 9.1**	− 7.2	− **9.2**
**2-(3,7-Dimethyl-octa-2,6-dienyl)-1,4-dimethoxy-benzene**	− 6.2	− 8.1	− 6.8	− 8.7
1,1′-2,2′-Bis[2,3-dimethylbenzoquinonyl]	− 7.0	− 5.8	− 6.7	− 7.1
**Ethyl cholate**	**− 7.8**	− 7.4	− 7.0	− 8.9
**Sorafenib(anticancer agent)**	**− 7.7**	**− 7.9**	**− 7.6**	**− 8.4**

Significant values are in bold.

**Table 7:** Molecular interactions of selected phytocompounds with target macromolecules.

Compound’s name	TNF-α		TGF-β R1		Bcl-2		iNOS	
	Interacting residues	Bond length	Interacting residues	Bond length	Interacting residues	Bond length	Interacting residues	Bond length
2-Methyl-6-(4-methylphenyl)hept-2-en-4-one	**TyrB151** TyrB59 TyrA119 TyrB119	**2.48** 3.62 3.58 3.77	**LysA232** IleA211 ValA219 AlaA230 LysA232 TyrA249 LeuA260 PheA262 LeuA278 TyrA282 LeuA340	**2.69** 3.70 3.69 3.57 3.56 3.59 3.20 3.81 3.59 3.74 3.54	PheA101 TyrA105 MetA112 LeuA134 ArgA143 PheA150 ValA153	3.68 3.79 3.73 3.72 3.96 3.66 3.63	TryA194 GlnA205 LeuA209 PheA369 TryA372	3.78 3.81 3.46 3.36 3.56
Phenol,2-methyl-5-(1,2,2-trimeth ylcyclopentyl)	**GlyA121** LeuB57 TyrB59 TyrA119 TyrB119	**2.91** 3.46 3.80 3.67 3.70	**TyrA249** LysA213 LysA232 LeuA260 LeuA340 AspA351	**1.99** 3.43 3.62 3.85 3.65 3.77	PheA101 TyrA105 AlaA146 GluA149	3.87 3.95 3.62 3.82	**AsnA370** TrpA194 ProA350 PheA369	**2.71** 3.42 3.71 3.36
Deoxyshikonin	LeuA120 TyrA59 TyrB59 TyrB151	3.20 3.59 3.80 3.88	**SerA280** **HisA283** **AspA281** IleA211 AlaA230 LysA232 LeuA260 Leu278 TyrA282 lEUA340	**2.70** **2.44** **2.40** 3.43 3.74 3.63 3.73 3.79 3.79 3.41	PheA109 MetA112 GluA133 LeuA134 PheA150 ValA153	3.40 3.75 3.71 3.60 3.66 3.56	**GluA377** TryA194 IleA201 LeuA209 PheA269 MetA374 TyrA489	**2.97** 3.81 3.89 3.61 3.72 3.60 3.65
2-(3,7-Dimethyl-octa-2,6-dienyl)-1,4-dimethoxy-benzene	TyrA59 TyrB59 TyrA119 TyrA151	3.54 3.81 3.55 3.78	IleA211 ValA219 LysA232 TyrA249 LeuA260 PheA262 LeuA278 LeuA340	3.97 3.74 3.35 3.60 3.71 3.33 3.63 3.71	PheA101 TyrA105 AspA108 LeuA134 AlaA146 PheA150	3.62 3.82 3.53 4.00 3.79 3.45	TryA194 AlaA197 ArgA199 PheA369 PheA488 TyrA489	3.75 3.73 3.53 3.48 3.73 3.67
Ethyl cholate	**TyrA59** **TyrB151** LeuA57 LeuB57 TyrA59 TyrB59 IleB155	**2.09** **2.43** 3.72 3.88 3.68 3.47 3.76	**IleA211** LysA213 ValA219 AlaA230 LysA232 LeuA260 LysA337 LeuA340	**2.85** 3.49 3.94 3.14 3.63 3.15 3.96 2.43	**GluA176** **TyrA177** PheA127 ValA131 TyrA177	**2.70** **2.88** 3.63 3.78 3.50	**ArgA199** **CysA200** LeuA125 ArgA199 ValA352 PheA369 TyrA489 TyrA491	**2.17** **2.29** 3.90 3.94 3.28 3.88 3.52 3.71

Highlighted residues: residues involved in hydrogen bond formation.

Other residues: residues involved in hydrophobic bond formation.

#### ADMET analysis of drug candidates

The detailed ADMET analysis of the top five compounds with the best hits is shown in Table 7. The literature survey indicated that the Ames test is important, and its positive value indicates the mutagenicity of that compound. All compounds showed a negative value that proved them non-mutagenic. Among these compounds, the water solubility of deoxy-shikonin was better, and ethyl iso-cholate showed the best absorption in the human intestine. Also, both these compounds were positive for the P-gp substrate. All selected compounds had higher GI absorption, an important criterion for drug entrance into the human body. These compounds are also predicted to have a penetration through the blood–brain barrier and might be effective for the treatment of neurological disorders. During drug metabolism, no one compound was metabolized by CYP2C9, CYP2D6, and CYP3A4. Three compounds were found to be inhibited by CYP1A2 and two were inhibited by CYP2C19. Only one compound was predicted as an inhibitor of herGII, while no compound was detected as an OCT2 renal substrate and hepatotoxic. The total clearance value of the testing phytochemicals varied as deoxy-shikonin exhibited the lowest value, and Phenol, 2-methyl-5-(1,2,2-trimethylcyclopentyl) indicated the greatest value cleared from plasma. Overall, these hit compounds have great potential to use as safe drugs in humans and animals. Other information is also given in Table 8.

**Table 8:** ADMET analysis of selected compounds using PkCSM software.

Pharmacokinetic parameters	2-Methyl-6-(4-methyl phenyl)hept-2-en-4-one	Phenol,2-methyl-5-(1,2,2-trimethyl cyclopentyl)	Deoxy-shikonin	2-(3,7-Dimethyl-octa-2,6-dienyl)-1,4-dimethoxy-benzene	Ethyl cholate
Solubility in water	− 4.454	− 4.496	− 3.786	− 5.761	− 4.734
Caco-permeability	1.458	1.607	0.928	1.796	1.032
Human intestinal absorption	1.458	90.21	91.339	94.197	97.702
Skin permeation	1.458	− 1.874	− 3.084	− 2.134	− 4.057
Substrate of P-glycoprotein	No	No	Yes	No	Yes
Permeation of blood–brain barrier	Yes	Yes	Yes	Yes	Yes
Gastrointestinal absorption (GI.)	High	High	High	High	High
Inhibitor of CYP1A2	Yes	Yes	No	Yes	No
Inhibitor of CYP2C19	No	No	Yes	Yes	No
Inhibitor of CYP2C9	No	No	No	No	No
Inhibitor of CYP2D6	No	No	No	No	No
Inhibitor of CYP3A4	No	No	No	No	No
Total clearance (volume of plasma cleared of a drug)	0.295	0.922	0.077	0.446	0.748
Renal OCT2 (optical coherence tomography) substrate	No	No	No	No	No
AMES test	No	No	No	No	No
Hepatotoxicity	No	No	No	No	No
Maximum tolerated dose in human	0.846	0.577	0.63	0.6	− 0.596
Acute oral rat toxicity (LD50)	1.843	2.18	1.662	1.873	2.045
Chronic rat oral toxicity	1.11	1.273	2.117	2.445	0.141
Skin sensititivity	Yes	Yes	No	Yes	No
Toxicity of* T. pyriformis*	1.945	1.933	0.824	2.857	0.401
Minnow toxicity	0.005	0.238	1.341	− 0.452	0.343
hERG I inhibitor	No	No	No	No	No
hERG II inhibitor	No	No	No	Yes	No

## Discussion

The Arnebia genus is a member of the Boraginaceae family. Its few species are found in the arid region of North Africa, but they are typically restricted to Asia. Ancient cultures employed the air-dried roots of *Arnebia nobilis* to treat wounds and fever amelioration^62^. From this genus, numerous secondary metabolites have been investigated for their biological activities^15^. But to our knowledge, the therapeutic potential of *A. nobilis* against hepatocellular carcinoma was still unexplored. During long-term conventional antitumor treatments, severe side effects, non-specificity, and drug resistance are big challenges. Therefore, recent research focuses on finding alternative natural treatment options with less or no adverse effects^63^. About 60% of marketed drugs are plant-based with effective pharmacological properties. In recent years, many novel anticancer drugs have been commercially derived from different plants, including vinca alkaloids, podophyllotoxin, taxanes, vincristine, and their derivatives^64^. Several studies reported various medicinal plants' antioxidative, cytotoxic, and apoptotic potential^65^. So, natural anticancer agents are safer than synthetic medicines that affect even normal cells^66^.

Recently, medicinal plants have been increasingly used in the treatment and management of inflammation-induced cancer due to the presence of diverse bioactive phytochemicals. According to our findings, both extracts showed the presence of alkaloids, flavonoids, glycosides, tannins, quinones, and phenols. Still, only saponins in the ethanolic extract and terpenoids in the n-hexane extract were observed (Table 2). In this respect, Chauhan et al. reported the presence of anthraquinones, straight-chain alkane, 4-hydroxybenzoic acid, and alkaloids in different solvents of *A. nobilis*. In another previous study, flavonoids, alkaloids, and glycosides were identified in the ethyl methyl ketone fraction of *A. nobilis*^16^. Mainly, flavonoids, alkaloids, saponins, and phenols have been found to have a wide range of anticancer actions, including modulating the activity of antioxidant enzymes, cell cycle arrest, induction of apoptosis and autophagy, reducing inflammation, inhibition of cancer cell proliferation and invasiveness^67–70^. Quinones contain a naphthalenic ring that is effective against human cervical carcinoma, liver, gastric, and breast cancer through stimulating autophagy and cell death^71^. In addition, cardiac glycosides were well-known to downregulate IL-8 and DNA topoisomerase I and II levels, prevent anoikis and reduce the number of target genes involved in inflammation to suppress carcinogenesis^72^. Moreover, terpenoids are also a major class of phytochemicals with positive anticancer effects by reducing the early stages of cancer development via induction of cell cycle arrest, minimizing inflammation, preventing cancer cell differentiation, and activating apoptosis^73^.

In our investigation, both extracts showed better antioxidant activity in a concentration-dependent manner (Table 3). It was observed that ANH extract showed a better scavenging potential of free radicals with IC_50_ values of 39.45, 64.44, and 90.88 µg/mL in DPPH, nitric oxide, and superoxide anion procedures, respectively. In comparison, the ethanol extract exhibited a better antioxidant activity with less IC_50_ value (91.27 µg/mL) in the hydrogen peroxide scavenging assay. Interestingly, n-hexane extract was found to be more antioxidative in most of the scavenging assays than ethanolic extract, which is consistent with the previous literature. This study is further supported by Jadid et al. reporting about the n-hexane extract of *P. retrofractum* fruit that scavenged free radicals more effectively than methanol and ethyl acetate extracts^74^. It was also observed that both extracts exhibited less antioxidant activity than ascorbic acid. In contrast to our results, another study demonstrated more scavenging activity of *A. nobilis* root extracts compared to conventional ascorbic acid^16^. The antioxidant potential of plant extracts was definitely due to the presence of therapeutically active phytochemicals. Our research revealed that *A. nobilis* has great potential as a reliable drug to treat several oxidative stress-related diseases.

Numerous research has revealed the anticancer potential of medicinal plants with several therapeutic natural chemicals. These medicinal components operate through various antitumor mechanisms of action but are commonly involved in apoptotic induction. In our study, cytotoxicity of *A. nobilis* extracts was observed in a concentration-dependent manner against HepG2 and BHK cells using an MTT assay (Fig. 2). These results were further confirmed by crystal violet assay (Fig. 4). The American cancer research institute has given the criteria of IC_50_ value should be less than 30 μg/mL to determine the cytotoxicity of a crude extract as a potent anticancer agent^75^. According to our findings, n-hexane extract and cisplatin (standard drug) proved to be more cytotoxic with IC_50_ values of 22.77 and 25.53 µg/mL, respectively, as their IC_50_ < 30 μg/mL in comparison to ethanol extract (IC_50_ of 46.74 µg/mL). No significant data is available about the cytotoxic activity of *A. nobilis* root extracts against HepG2 cells.

Different studies reported the strong antioxidative and antiproliferative activities of other members of this family (Boraginaceae) against various cancers^76^. For example, Asghar et al. showed that the petroleum ether and an aqueous fraction of *Onosma hispidum* Wall, Exhibiting a better cytotoxic effect against HepG2 (hepatocellular carcinoma) cell line^77^. Similarly, Demir et al. evaluated the antiproliferative potential of phenolic compounds in *Onosma armeniacum* root extract against colon, lung, and human liver cancer cell lines^78^. The main objective of cancer therapeutics is to target specifically malignant cells without destroying normal cells, that is the main limitation of chemotherapeutic drugs. In the present study, both plant extracts showed cytotoxicity against HepG2 cells without damaging the normal cells, while cisplatin (a platinum-based anticancer drug) induces an antitumor effect through apoptotic stimulation in cancer as well as in normal (non-cancerous) cells, these outcomes are inconsistent with the previous study^79^. So, this plant can potentially treat liver cancer without disturbing normal cells. Different chemotherapeutic drugs induce cell death in cancer cells by using discrete modes of action to inhibit carcinogenesis^80^.

Similarly, natural products are well known for targeting cancer cell proliferation by initiating various apoptotic pathways^81^. In our study, treating malignant cells (HepG2 cells) with both extracts and standard drugs at their IC_50_ concentrations, a higher percentage of cell death was observed in ANH and cisplatin-treated cancer cells by promoting apoptosis than in ANE-treated cells (Fig. 5). No data is available about the apoptotic activity of *A. nobilis* against liver cancer cells. But, another specie of Borignaceae family, *Onosma bracteata* showed induction of cell death in MG-63 cells by enhancing the expression of p-53 and decreasing the level of Bcl-2, cyclin-E, cyclin-dependent kinase 2 (CDK2), and mortalin^82^. While cisplatin (standard drug) is responsible for inducing DNA damage and P53-mediated apoptosis in cancer cells was already reported in the literature^83^. To the best of our knowledge, this is the first-hand report about the apoptotic potential of *A. nobilis* against malignant hepatic cells. So, this plant might be a promising anticancer candidate with an apoptotic mode of action in targeting specifically the malignant cells without affecting normal cells.

Moreover, the n-hexane extract was further selected for GCMS analysis based on its higher antioxidative and anticancer potential. GCMS analysis detected several bioactive compounds in the n-hexane fraction (Table 4). Out of thirty-five bioactive compounds, only fourteen were found to be biologically active (Table 6). Among these compounds, deoxy-shikonin had the highest peak area percentage and was already isolated from different plants of the Borignaceae family. This compound exhibited antibacterial, antifungal, wound healing, and antitumor properties^84^ but its underlying mechanism in inflammation-induced HCC was still unclear. Thymol is a phenol with antibacterial, anti-inflammatory, anti-mutagenic and radioprotective properties^85^. Another compound, 9,12-Octadecadienoic acid, ethyl ester is a fatty acid ester with hepatoprotective, antimicrobial and anticoronary activities^86^. In general, it was observed that bioactive phytoconstituents identified by GCMS in medicinal plants have a wide range of biological activities^87,88^. Therefore, the natural components screened from ANH extract may have a significant role in the pharmacological and biological activities, additional research should be required to investigate them. Nowadays, bioinformatic tools are extensively used in the prediction of drug-like bioactive molecules during drug discovery^89^. These computational approaches were used to predict the therapeutic effects of phytocompounds, which were later confirmed by in-vitro and in-vivo studies^5^.

Researchers may be able to develop novel alternative therapies by better understanding the mechanism of action of phytochemicals interacting with targets, to block or activate proteins and enzymatic pathways for treating a particular disease^90^. In the present study, the screening of drug-like compounds was done by following Lipinski’s rules and satisfying toxicological parameters (Table 5). A compound that goes beyond these limits is unlikely to be further investigated as a drug because it may lose important properties associated with absorption, metabolism, distribution, and excretion^91^. Out of thirty-five compounds detected in n-hexane extract, only fourteen compounds were biologically active according to these limitations. The biological significance of selected compounds were compared with the standard drug (Sorafenib) given in Table 5. As we know that the tumor microenvironment (TME) is an intricate system that promotes cancer from initiation to metastasis and is continuously regulated by cellular metabolism, genetic changes, epigenetic factors, and dysfunctional oncogenic signaling. The backbone of this complex tissue milieu was built up by extracellular matrix (ECM) and contains a variety of stromal cells, fibroblasts, innate and adaptive immune cells, as well as non-cellular substances including the pro- and anti-inflammatory cytokines, signaling proteins, and growth factors^92^. In inflammation-induced hepatocellular carcinoma, the upregulated levels of TNF-α, TGF-β, TGFβ R1, Bcl2, and iNOS were frequently observed^22,93–95^. Increased concentration of nitric oxide synthase is responsible for increasing the level of RNS (reactive nitrogen species), which in turn upregulate the oxidation and nitration of proteins, which may lead to chronic inflammation^96^. Similarly, TNF-α is a crucial mediator and an important part of the cancer-linked inflammatory network. TNFα-related NF-κB activation contributes to the expression of antiapoptotic and antioxidant genes, blocking cell death via deactivating the JNK pathway, which facilitates cancer cell proliferation^97^. Additionally, a growing number of studies have shown that TGFβ1 and TGFβR1 activate NF-κB, JAK/STAT3 signaling pathways and microRNAs (miR-133b), to promote the proliferation, migration, and epithelial-mesenchymal transition (EMT) in tumor cells^93^. Notably, TGFβ1 and TGFβR1 are two important members of the TGFβ-signaling pathway that are significantly expressed in many tumors, including colon cancer, gastric cancer, breast cancer, and hepatocellular carcinoma^98^. Moreover, overexpression of Bcl-2 (antiapoptotic protein) participates in tumor formation and the development of multidrug resistance by inhibiting apoptosis and regulating cell proliferation. Another study showed that elevated levels of mRNA and Bcl-2 proteins were observed in HCC tissues^94^. Therefore, inhibiting the Bcl-2 protein is a novel therapeutic approach to overcome the resistance of tumor cells against apoptosis.

Consequently, in the current study, we selected these basic targets (TNF-α, TGF-βR1, Bcl-2, and iNOS) as most of the proteins have a significant role in inflammation-related liver carcinogenesis. Among fourteen bioactive molecules, only five top scorer compounds showed better inhibitory effects against selected cancer-causing targets. Among these compounds, deoxyshikonin, ethyl cholate, and 2-Methyl-6-(4-methylphenyl)hept-2-en-4-one exhibited better results with more interactions and docking scores against most of the targets. At the same time, the remaining compounds also depicted satisfactory results compared to sorafenib (Tables 6 and 7). So, the ADMET properties of these top scorers were found to be satisfactory as displayed in Table 8.

Thus, these best-hit biomolecules derived from the n-hexane extract of *A. nobilis* showed better modulation of oxidative stress, anti-inflammatory, anticancer, and apoptotic potential to define their promising role in developing the multiple-targeted antineoplastic drugs. So, these compounds are proven good candidates to be a part of different anticancer therapeutic regimens against HCC but should be further explored using different in-vivo studies.

## Conclusion

The primary goal of the current work was to demonstrate the strong antioxidant, cytotoxic and apoptotic potential of *A. nobilis* root extracts. The n-hexane extract showed better results and underwent through GCMS analysis to profile pharmacologically active phytochemicals. Fourteen compounds were selected for molecular docking based on toxicological and drug-like parameters. Among these, five compounds showed better results, but overall, deoxy-shikonin, ethyl cholate and 2-Methyl-6-(4-methylphenyl)hept-2-en-4-one showed the best hits against most of the target macromolecules to inhibit hepatocarcinogenesis via inhibiting inflammation, promoting apoptosis, and reducing angiogenesis in malignant cells. According to our knowledge, most of the biological activities of *A. nobilis* are not well-documented previously. Thus, this plant, especially its n-hexane extract, has great potential to develop into a safe, specific, and effective medication for preventing and managing hepatocellular carcinoma. For new medication formulations, additional in-vivo studies are required to authenticate its anticancer role via exploring various underlying mechanisms involved in inflammation-induced liver carcinogenesis.

## Data Availability

All of the data produced or generated during the study has been given in the manuscript.
